# Invasive alien insects represent a clear but variable threat to biodiversity

**DOI:** 10.1016/j.cris.2023.100065

**Published:** 2023-07-14

**Authors:** David A. Clarke, Melodie A. McGeoch

**Affiliations:** a Department of Environment and Genetics, La Trobe University, Victoria 3086, Australia; b Securing Antarctica's Environmental Future, La Trobe University, Victoria 3086, Australia

**Keywords:** Invasive alien species, Environmental impacts, Knowledge shortfalls, Impact assessment

## Abstract

Invasive alien insects are an important yet understudied component of the general threat that biological invasions pose to biodiversity. We quantified the breadth and level of this threat by performing environmental impact assessments using a modified version of the Environmental Impact Assessment for Alien Taxa (EICAT) framework. This represents the largest effort to date on quantify the environmental impacts of invasive alien insects. Using a relatively large and taxonomically representative set of insect species that have established non-native populations around the globe, we tested hypotheses on: (1) socioeconomic and (2) taxonomic biases, (3) relationship between range size and impact severity and (4) island susceptibility. Socioeconomic pests had marginally more environmental impact information than non-pests and, as expected, impact information was geographically and taxonomically skewed. Species with larger introduced ranges were more likely, on average, to have the most severe local environmental impacts (i.e. a global maximum impact severity of ‘Major’). The island susceptibility hypothesis found no support, and both island and mainland systems experience similar numbers of high severity impacts. These results demonstrate the high variability, both within and across species, in the ways and extents to which invasive insects impact biodiversity, even within the highest profile invaders. However, the environmental impact knowledge base requires greater taxonomic and geographic coverage, so that hypotheses about invasion impact can be developed towards identifying generalities in the biogeography of invasion impacts.

## Highlights

• Environmental impacts of invasive alien insect species are understudied.
• We assessed the environmental impacts of 352 alien insect species.
• There was high intra-and interspecific variation in impact mechanism and severity.
• Evidence for impact severity-range size relationship but not island susceptibility.
• Results highlight knowledge gaps to guide future environmental impact research.

## 1 Introduction

Insects are one the most abundant and species rich animal groups on land (Stork, 2018). Unsurprisingly, there are also many insect species with populations established outside of their native range; the total estimated number of alien insects is as high as one quarter of the potential source pool of insect species (Liebhold et al., 2018; Seebens et al., 2018). Many insect introduction events are unintentional and a consequence of international trade (Gippet et al., 2019). This is especially true for agricultural and horticultural trade that harbors insects as stowaways, a consequence of the close association between floral commodities and insect occurrence (Liebhold et al., 2012, 2016). Not all introduced insect species are expected to negatively affect their recipient environments (Blackburn et al., 2011), although the probability that an introduced species will have negative consequences has been shown to increase with the number of introduction events (Cassey et al., 2018; Simberloff, 2009). This propagule pressure is expected to continue for both insects and other groups and understanding the consequences of invasion for biodiversity and ecosystems remains key to managing natural and agricultural environments into the future (Seebens et al., 2021).

Alien insects are well-known for their socioeconomic impacts (Vilà et al., 2010), including economic loss (Bradshaw et al., 2016; Renault et al., 2022) from decreased crop yields (Fried et al., 2017) and management costs (Barbet-Massin et al., 2020; Hoffmann and Broadhurst, 2016), through to threatening food security (Paini et al., 2016) and human health (Juliano and Lounibos, 2005; Mazza et al., 2014). However, their environmental impacts - realized and potential - are of equal concern. The cumulative effect of alien insects can cascade through food-webs, mediated via multiple direct and indirect interactions, and impact environmental processes from populations to ecosystems (Noriega et al., 2018; Yang and Gratton, 2014). There are many examples of such damaging impacts, particularly by Hymenoptera (bees, wasps, ants, sawflies (Beggs et al., 2011; Hill et al., 2016; Inoue et al., 2007; Kenis et al., 2009; Snyder et al., 2007). Recent reviews have considered effects on native insect communities (Fortuna et al., 2022), and socioeconomic and environmental impacts of ants in particular (Gruber et al., 2023). However, a general understanding of the range, features and comparative severity of the specific environmental impacts (impact mechanisms) of invasive alien insects is still missing (Clarke et al., 2021; Lapin et al., 2021, Pyšek et al., 2020).

One method for synthesizing available information on invasion impacts, in a useful and targeted form, is ‘environmental impact assessment’. This tool is used to guide the development of evidence-based risk assessments (Roy et al., 2018). The method of semi-quantitatively processing available information is particularly useful for insects because they are often under-represented in biodiversity databases (Troudet et al., 2017) and have taxonomic, distribution and abundance-based knowledge shortfalls (Hortal et al., 2015). Information on environmental impacts, which are necessarily informed by species population and life history trait information, is similarly poor for alien insects (Crystal-Ornelas and Lockwood, 2020, Renault et al., 2022). Therefore, using existing information along with a structured protocol to classify the severity of impacts so far realized (i.e., impact history) for alien species improves the potential for predicting future impacts (Kulhanek et al., 2011), and facilitates triage in species-based prioritization (McGeoch et al., 2016). Such evidence-based risk assessments are important for the appropriate prioritization of invasive species for management and prevention (Carboneras et al., 2018; Genovesi et al., 2014; Roy et al., 2018).

Here we assess the environmental impacts of invasive alien insects at both national and global scales. We individually assess and then collate the information across species to quantify geographic and taxonomic variation in the severity of impacts, and the mechanisms by which invasive alien insects impact native environments. We test four hypotheses about invasive alien insect impacts, the first two to better understand bias in impact evidence availability and the second two to test biogeographic hypotheses of insect invasion.

H1: The environmental impacts of invasive alien insects of socioeconomic concern are better known than the environmental impacts of invasive alien insects that do not have socioeconomic impacts. This is because species of socioeconomic concern are expected to receive most research attention overall (Essl et al., 2016; Vilà et al., 2010), which results in greater attention also being paid to their environmental impacts (McGeoch et al., 2015).

H2: General understanding of the impacts of invasive alien insects is biased by more research on some taxonomic groups than on others. General conclusions about invasive species impacts are likely to be based on knowledge biased towards particular high-profile species or taxonomic groups (Watkins et al., 2021). Hymenoptera have previously been suggested to be better studied than other insect orders (Kenis et al., 2009).

H3: The more widespread an alien insect species is in its introduced range, the more likely it is to encounter conditions conducive to the realization of it effecting a severe environmental impact. This will result in a positive relationship between the introduced geographic range of a species and its maximum environmental impact. Geographic range size (as a measure of total area occupied) is considered one of three key factors that determine the environmental impact of an alien species (the others being abundance and per capita effect, Parker et al. 1999). Examinations of the abundance-impact relationship have provided evidence for the influence of a species abundance on their environmental impact (Bradley et al., 2019), however, examinations of a range size-impact relationship are lacking. Here, we classify impacts independently of range size and propose and test this impact hypothesis for the first time.

H4: Alien insect species are “more likely to become established and have major ecological impacts on islands than on mainlands” (Jeschke, 2008), i.e. does the island susceptibility hypothesis hold for insects? The island susceptibility hypothesis is that island ecosystems experience more harmful impacts, and harmful impacts more often, than mainland locations because island biota are more species poor and less competitive (Enders et al., 2020, Jeschke, 2008). In both birds and mammals, islands are well-known to be highly susceptible to negative biodiversity impacts from invasive alien species (Doherty et al., 2016; Evans et al., 2016), and although evidence varies (Sol, 2000) harmful environmental impacts from alien birds have been shown to be higher for island-located impacts than impacts elsewhere (Evans et al., 2016). This hypothesis has not been thoroughly tested for insects.

## 2 Methods

### 2.1 Species pool assessed

Impact assessments were carried out on a subset of the 590-insect species of environmental concern identified in Clarke et al. (2021). From this species pool of 590, Clarke et al. (2021) used 100 species in a pilot assessment to test and refine the impact assessment protocol. We re-assessed these 100 species here so that impact assessments for all species used up-to-date information and followed the same process. Additional species were then selected for assessment. For orders with < 20 species (*n* = 13 orders) in the species pool of 590, all species in the order were assessed. For orders with > 20 species present (*n* = 6), 20 species per order were randomly selected. The final set of species assessed for the purpose of testing the four hypotheses included 352 species from 19 taxonomic orders, was thus considered to be taxonomically representative of insect species considered to have negative environmental impacts somewhere in their introduced range.

### 2.2 Impact assessments

Environmental impacts of alien insects were assessed using the Environmental Impact Classification for Alien Taxa (EICAT) protocol (Blackburn et al., 2014; Hawkins et al., 2015; IUCN, 2020), and following the recommendations for applying this method in Clarke et al., (2021). In addition, rather than using only the global maximum impact severity (i.e., a single impact severity score), for the purpose of quantifying variation in both how and where particular alien species are negatively affecting the environment, we included information on each recorded instance of environmental impact evidence. EICAT is a semi-quantitative method that uses available evidence to classify the negative environmental impacts of alien species according to their severity and mechanism of impact, i.e., Minimal Concern (MC, impacts on native taxa negligible), Minor (MN, no evidence for a decline in population sizes of native taxa), Moderate (MO, impact native species population sizes but no evidence of local apparent extinction), Major (MR, reversible local extinction of one or more native taxa) and Massive (MV, irreversible local extinction of one or more native taxa) (for full descriptions of categories used see Clarke et al. (2021)).

Differential literature use, for example due to variable search strategies, is a primary cause of assessment outcome differences among independent assessors (Clarke et al., 2021). Therefore, taking a systematic approach to searching the available impact evidence can decrease uncertainty in the assessment outcome and increase transparency. Web of Science (WoS, www.webofknowledge.com) was the primary database used for literature searches. However, it is important to note that we only searched and used information from the published, peer-reviewed literature, and did not consider impact information that may be available in, for example, the grey literature. Literature searches used *Web of Science All Databases* (not only *Core Collection*) with the timespan selected as *All years*. For a given species, the search string consisted of the species binomial (or trinomial in the case of subspecies) name with the Boolean operator OR used to separate all known synonyms. Accepted names and synonyms were determined using the Global Biodiversity Information Facility (GBIF) taxonomic backbone. Using only species names can result in many search returns. Although this leads to the return of many irrelevant publications, consequently increasing the time taken to complete an assessment, it also decreases the likelihood of missing relevant information that contains a more nuanced form of impact evidence. Additionally, this species-specific approach to searching the literature enables a more complete picture of the state of alien insects and their environmental impacts, as much impact research is biased toward a subset of well-studied species (Cameron et al., 2016; Crystal-Ornelas and Lockwood, 2020; Kenis et al., 2009). Publications warranted examination if the title and/or abstract provided any indication that the alien insect species in question was negatively affecting the native environment in a location outside of the species native range. Non-English publications that were deemed potentially relevant based on the translated title provided by WoS were translated using Google Translate to confirm their relevance for inclusion and thus reduce, to some extent, a bias toward English-language publications which can omit important information (Angulo et al. 2021, Nuñez and Amano, 2021). Following the WoS searches, the same search strings were included in Google Scholar with the first 50 returns checked for titles not returned in the WoS search. To ensure transparency, all literature search information, including dates of search, terms used, and the search outcomes, were recorded and is available online, along with all code for replicating the analyses (https://doi.org/10.5281/zenodo.7508641).

Publications included for assessment were those in which there was evidence of an introduced insect species negatively affecting the native biodiversity/environment. An example of a study type *not* included is one in which native species were negatively affected by human actions related to management of the alien species under assessment, e.g., a chemical spray intended for the alien species that negatively affects native species. Similarly, if humans introduced a biocontrol agent to control the alien species under assessment, and that biocontrol agent negatively affects the native environment, this was also not considered (it would, however, be evidence under an assessment of the biocontrol agent itself). Furthermore, the focus was on *what* was affected, not *where* it was affected. For example, whilst evidence of an alien insect damaging an agricultural crop was not included, evidence for the same insect negatively affecting a native species *within* an agricultural crop was included.

Complementing the literature searches were two other secondary sources of information. First, potentially relevant studies that were cited by an examined paper but were not themselves included in the literature search returns were also assessed. Second, Center for Agriculture and Bioscience International (CABI) species datasheets from both the Crop Pest Compendium (CPC) and Invasive Species Compendium (ISC) were examined for any additional information on each species. The presence or absence of both CPC (pest) and ISC (invasive species) datasheets was also recorded for testing the hypothesis that environmental impact research is biased toward socioeconomic pests.

### 2.3 Data analysis

Species were considered a socioeconomic pest if their CABI CPC datasheet listed them as such; datasheets were therefore used as a proxy for socioeconomic pest status. If a species did not have “pest” listed on their CABI CPC datasheet, or a datasheet was absent, they were not considered a socioeconomic pest. A Wilcoxon rank sum test was performed to assess if research effort, here defined as the number of WoS search returns, is a function of socioeconomic pest status, using the *rstatix* R package (Kassambara 2021). Poisson generalized linear mixed models (GLMM) were used to test the hypothesis that insect species of socioeconomic concern are researched more for their environmental impacts than those that are not of socioeconomic concern (H1). Number of environmental impact publications was the response variable of interest, the logarithm of the number of search returns and socioeconomic pest status were fixed effects, and scientific name and taxonomic order of each species were the random effects. Number of total search returns per species was included to account for the fact that there was high variation in research effort across species assessed. GLMMs were performed using the *lme4* R package (Bates et al., 2015).

Poisson generalised linear models (GLM), by way of a loglinear analysis, were used to test the hypothesis that environmental impact information availability varies according to taxonomic order (H2). Loglinear models were fit to a 2 × 2 contingency table cross-classifying 336 insect species on the categorical response variables taxonomic order and environmental impact information availability. GLMs were performed using the base R *stats* package.

Assessing the relationship between impact severity and alien geographic range would ideally use extent of occurrence (EOO) (Gaston, 1991) as the measure of range size. However, given the taxonomic and spatial biases present in species occurrence data (Beck et al., 2014; Troudet et al., 2017; Hughes et al. 2021), we chose instead to use the number of countries listed for a species in the Global Register of Introduced and Invasive Species (GRIIS), which includes verified records of presence at a country scale, as a proxy for alien geographic range (Pagad et al., 2018, 2022). Cumulative link (mixed) models (CL(M)M) were used to test the hypothesis that there is a positive relationship between impact severity and introduced geographic range size (H3). Global maximum impact severity, i.e., one record per species, was the response variable. As such, only species with evidence of impact were used in the analysis (i.e., excluding any Data Deficient species). For each species, the number of countries with established alien populations, the logarithm of the combined area of the associated countries, and the combined number of shipping ports across the associated countries were included as fixed effects. For the mixed model, taxonomic order was included as a random effect. CL(M)Ms were performed using the *ordinal* R package (Christensen 2019). Country areas were calculated using the *sf* R package (Pebesma, 2018) after reprojecting the GADM shapefiles (Hijmans et al., 2010) to a Mollweide projection. Shipping port data was obtained from Natural Earth (https://www.naturalearthdata.com/downloads/10m-cultural-vectors/ports/).

Under the EICAT framework, impact severities of Moderate (MO) or higher are considered “harmful” impacts (IUCN, 2020). As such, impact severities were converted to a binary variable where severities >= MO considered harmful and severities < MO not harmful. To test the hypothesis that harmful impacts occur more often on islands than on mainland locations (H4), binomial GLMMs were used including the binary impact severity variable as the response. Landmass type, logarithm of the distance to the equator and logarithm of landmass area were included as fixed effects, and species name included as a random effect. All available environmental impact evidence was included, thus using species names as a random effect accounts for the fact that some species have more available evidence than others. As above, areas and distances were calculated using the *sf* R package after reprojecting the GADM shapefiles to a Mollweide projection.

## 3 Results

**Fig. 1:**
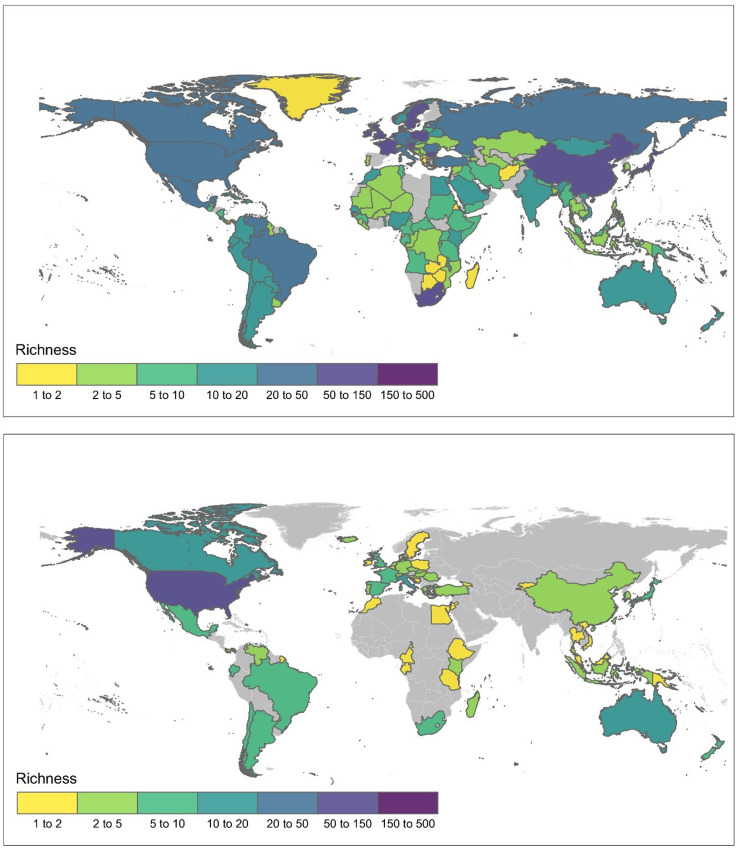
A. Global distribution of all alien and invasive insect populations known to harm the environment (*n* = 7049) at a country scale. B. Number of alien insect species with published evidence of environmental impact per country. Country boundary data from the Global Administrative Areas (GADM) database (Hijmans et al., 2010).

Evidence of environmental impact was available for 125 of the 352 species assessed, with approximately two thirds (*n* = 211; 59.9%) of species assessed as Data Deficient (Tables 1 and S1). Quantity of impact evidence among species varied widely, with a mean of 4.9 ± 7.1 SD impact publications per species (Figs. S1 and S2). The relatively high standard deviation reflects the presence of a small number of disproportionately well studied species (*Harmonia axyridis, Philornis downsi, Adelges tsugae, Apis mellifera, Bombus terrestris, Linepithema humile, and Solenopsis invicta*) with 15 to 50 publications demonstrating evidence of impact (Fig. S1, Table S2). Similarly, the number of species per country with evidence of impact was disproportionately higher in North America and Australia than elsewhere (Fig. 1A). Of the species assessed, Hymenoptera had the highest representation of species with impact evidence (*n* = 59) followed by Coleoptera (*n* = 17) (Table 1). There was no evidence of populations established outside of their native range for 16 of the 352 species assessed and these species are therefore not considered to have alien populations although they have been referred to as invasive in the literature (subsequently denoted NA) (Table 1).

**Table 1:** The number of species assessed in each insect order and the percentage for which at least one item of evidence on environmental impact was found. Parentheses include percentages of species assessed from species pool (Assessed), and species assessed with impact evidence (Evidence). Although three species of Trichoptera were initially included from the species pool based on them being referred to as ‘invasive’ in literature, all three were concluded to have no populations established outside their native range (i.e. NA).

Order	Assessed	Evidence	Species pool
Hymenoptera	98 (77%)	59 (60%)	128
Coleoptera	50 (34%)	17 (34%)	146
Hemiptera	49 (60%)	16 (33%)	81
Diptera	35 (80%)	14 (40%)	44
Lepidoptera	31 (55%)	8 (26%)	56
Blattodea	9 (100%)	5 (56%)	9
Mantodea	2 (100%)	2 (100%)	2
Siphonaptera	4 (100%)	1 (25%)	4
Thysanoptera	10 (100%)	1 (10%)	10
Dermaptera	17 (100%)	1 (6%)	17
Psocodea	20 (33%)	1 (5%)	66
Orthoptera	2 (100%)	0 (0%)	2
Phasmatodea	6 (100%)	0 (0%)	6
Zygentoma	6 (100%)	0 (0%)	6
Ephemeroptera	2 (100%)	0 (0%)	2
Odonata	1 (100%)	0 (0%)	1
Embioptera	5 (100%)	0 (0%)	5
Neuroptera	2 (100%)	0 (0%)	2
Trichoptera	3 (NA)	0 (NA)	3
Total	352	125	590

### 3.1 Impact mechanism, severity and confidence

**Fig. 2:**
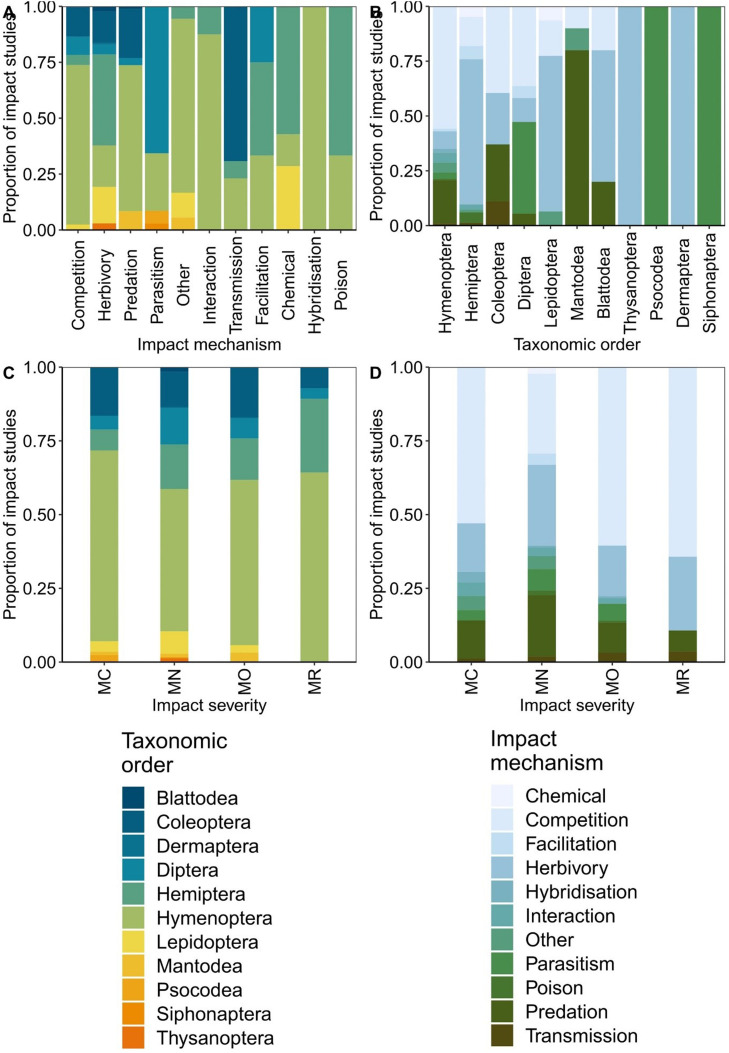
Evidence of the environmental impact of invasive alien insect species. Number of impact studies for: A. each environmental impact mechanism, per taxonomic order, B. each taxonomic order, per impact mechanism, C. each impact severity category, per taxonomic order and D. each impact severity category, per impact mechanism. Impact severity categories are Minimal Concern (MC, impacts on native taxa negligible), Minor (MN, no evidence for a decline in population sizes of native taxa), Moderate (MO, impact native species population sizes but no evidence of local apparent extinction), Major (MR, reversible local extinction of one or more native taxa) (there were no species assessed as having a Massive (MV) impact, irreversible local extinction of one or more native taxa) (for full descriptions of categories used see Clarke et al. (2021)). See Figs. S5 and S6 for a more detailed examination of the variation in impact mechanisms and severities.

Evidence of environmental impact was found on 11 impact mechanisms. Competition was the most frequently attributed impact mechanism across all instances of impact evidence (*n* = 244), followed by herbivory (*n* = 135), predation (*n* = 95), and parasitism (*n* = 35) (note a species may have evidence of impact for multiple mechanisms). As expected, dominant mechanisms differed across orders; for example, all cases of environmental impact via hybridization were associated with Hymenoptera, although overall hybridization was not amongst the most common mechanisms by which Hymenoptera have an environmental impact (Figs. 2, S3, S4). Similarly, although transmission of disease was attributed to Coleoptera more so than other orders, disease transmission was associated with only four of the 17 beetle species (Figs. 2, S3, S4).

The most common impact severity category attributed to species across all impact evidence was Minor (*n* = 317). This was followed by Moderate (*n* = 157), Minimal Concern (*n* = 85), and Major (*n* = 28), with no instance of a Massive impact (irreversible extinction) attributed. Hymenoptera had the highest proportion of recorded evidence for each category of impact severity (Figs. 2, S3, S4). However, variability in impact severity was also highest in the Hymenoptera (Figs. 2, S3, S4). The largest proportion of impact severity classifications per taxonomic order was also Minor (except Mantodea where it was Moderate) and no species of Blattodea, Dermaptera, Siphonaptera, and Thysanoptera had impacts in higher severity categories.

**Fig. 3:**
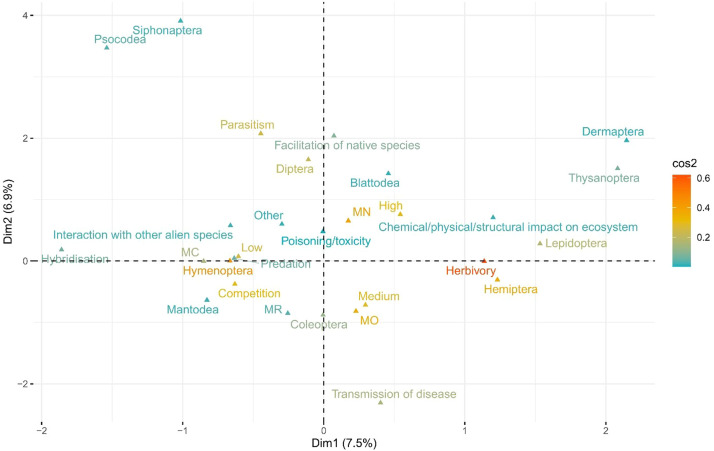
Multidimensional relationships between insect order, impact mechanism, impact severity, and confidence rating (Low, Medium, High) for each instance of environmental impact evidence, providing evidence of the context dependence of environmental impacts. Biplots of the first two dimensions of a multiple correspondence analysis (MCA). Levels of each categorical variable with distance between each variable level corresponding to similarity in profiles. Little to no relationship exists among the four variables, with the exception of some expected grouping such as herbivory and Hemiptera or competition and Hymenoptera. Impact severities are: Minimal Concern (MC), Minor (MN), Moderate (MO) and Major (MR).

Confidence ratings for each assessment of an individual piece of impact evidence varied from Low (*n* = 349) to Medium (*n* = 284) and High (*n* = 234). There was little to no correlation among taxonomy, impact mechanism, impact severity and confidence, with most points located around the origin of the first two ordination dimensions that collectively explained only 14.4% of the variation in the impact data (Fig. 3).

### 3.2 Pest status and data availability

When all species were pooled there were more WoS search returns for species with socioeconomic pest status than those without (*U* = 9597, *n_1_* = 180, *n_2_* = 172, *p* < 0.001, Fig. S5). The effect size of *r* = 0.33 denotes a moderate effect (Sullivan and Feinn, 2012) of socioeconomic pest status on WoS search returns. Socioeconomic pest status had a significant effect on the number of environmental impact publications specifically only by way of an interaction with the independent measure of research effort (logarithm of the WoS search returns). Species considered socioeconomic pests had less environmental impact evidence relative to non-socioeconomic pests, when accounting for research effort. However, there was high variation across the random effects of insect species (*σ^2^* = 1.699) and to a lesser extent taxonomic order (*σ^2^* = 0.6389).

Under the assumption of independence between taxonomic order and evidence of environmental impact, the estimated odds ratio (0.59) for a species of any given taxonomic order having evidence of environmental impact, relative to being data deficient, was significant (*p* < 0.0001). However, including an interaction term revealed strong evidence of an association between taxonomic order and the number of species assessed as Data Deficient. The probability that the independence model performed as well as the model containing an interaction between taxonomic order and assessment outcome (DD vs Not DD), if there was no association, was small (χ^2^ = 69. 454, *df* = 17, *p* < 0.0001). Under this model, the estimated odds ratio of 1.25 for any given taxonomic order having evidence of environmental impact relative to being data deficient was not significant (*p* = 0.74). For 16 taxonomic orders, the proportion of Data Deficient species was greater than non-Data Deficient (Table 2). Exceptions to this result were the Blattodea, Hymenoptera, and Mantodea where the opposite was found (Fig. S6). There was, however, high variation in the proportion of species assessed as Data Deficient across orders – ranging from all to none (Fig. S6).

**Table 2:** Number and percentage of species with environmental impact information was similar overall for species that are and are not considered socioeconomic pests. However, this pattern did not hold for some taxonomic orders. For example, the availability of environmental impact information differed depending on socioeconomic pest status for Coleoptera and Diptera. Non-socioeconomic pests had fewer data deficient (DD) species than pest species. Orders with one species or less across all categories are grouped as “Other”.

Taxonomic Order	Data deficient		Data available	
	SE pest	Non-SE pest	SE pest	Non-SE pest
Blattodea	2 (40%)	2 (50%)	3 (60%)	2 (50%)
Coleoptera	24 (75%)	8 (47%)	8 (25%)	9 (53%)
Dermaptera	7 (88%)	3 (100%)	1 (12%)	0 (0%)
Diptera	10 (77%)	10 (50%)	3 (23%)	10 (50%)
Hemiptera	27 (66%)	6 (66%)	14 (34%)	3 (34%)
Hymenoptera	11 (34%)	28 (42%)	21 (66%)	38 (58%)
Lepidoptera	17 (71%)	2 (66%)	7 (29%)	1 (33%)
Psocodea	2 (100%)	16 (94%)	0 (0%)	1 (6%)
Thysanoptera	8 (89%)	1 (100%)	1 (11%)	0 (0%)
Other	3 (75%)	24 (86%)	1 (25%)	4 (14%)
Total	111 (66%)	100 (60%)	58 (34%)	67 (40%)

### 3.3 Geographic range and impact severity

**Fig. 4:**
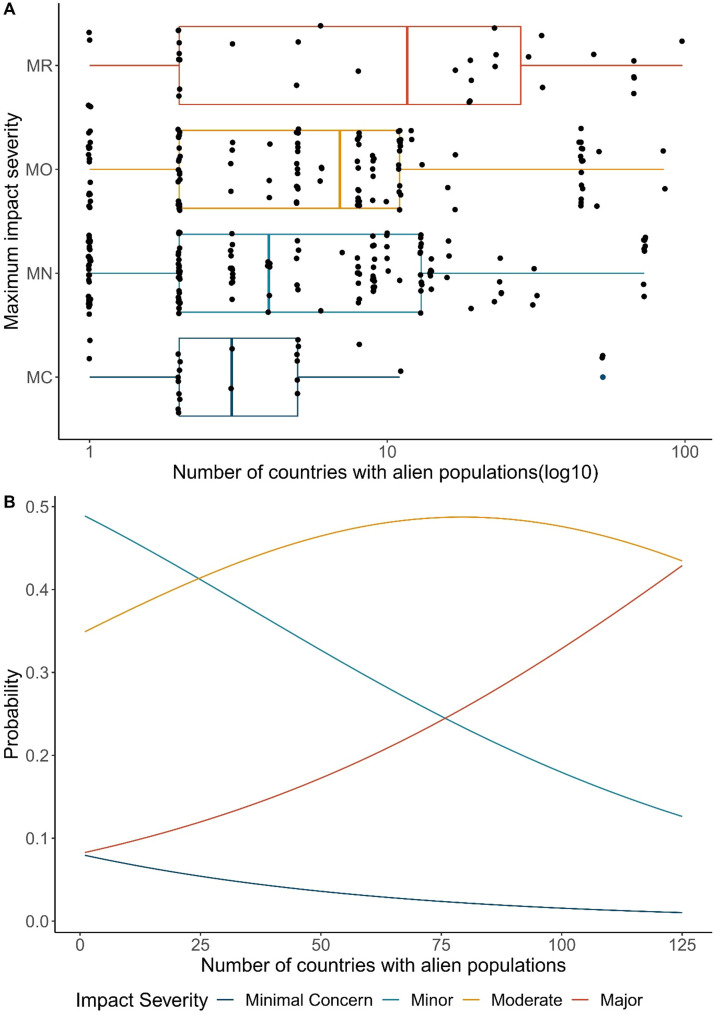
Global maximum impact severity for invasive alien insects is a function of alien geographic range. A. Alien geographic range variation (number of countries with alien populations) for species within each maximum impact severity classification. Most species had a maximum impact of Minor or Moderate severity. Impact severity categories are Minimal Concern (MC, impacts on native taxa negligible), Minor (MN, decreased performance of native taxa but no evidence of population decline), Moderate (MO, native species population declines but no evidence of local apparent extinction), Major (MR, reversible local extinction of native taxa) (there were no species assessed as having a Massive (MV) impact, irreversible local extinction of one or more native taxa) (for full descriptions of categories used see (Clarke et al., 2021). B. The relationship between geographic range and environmental impact severity of alien insects. The probability that the global maximum realized impact of an alien insect species falls in the most severe category (Major) is higher for those species established in the most countries, and lower for those species established in fewer countries.

Most countries in the world have at least one of the 590 alien insect species included in the initial source pool (Fig. 1A). Countries in Asia, Western Europe and Southern Africa have the most species in this pool representing known alien insects of environmental concern. The geographic distribution of populations with evidence of impact is patchier (Fig. 1B). Backward stepwise model selection by AIC revealed that neither random or fixed effects of total country area and number of shipping ports were important. The optimal model included alien geographic range (using number of country records per species in GRIIS) as the sole predictor of environmental impact severity (*χ^2^* = 5.8604, *df* = 1, *p* = 0.01549). There was also no evidence of non-proportional odds (*p* = 0.7291) nor scale effects (*p* = 0.4436). For a given insect species, the probability that its maximum impact severity will be Major increases as the number of countries with established populations of that species increases (Fig. 4). For every new country with an established population, the odds of the maximum impact being more severe are multiplied by 1.03, i.e., a three percent increase (95% CI 1.01, 1.05). In contrast, the probability that the maximum impact severity of a species will be Minimal Concern or Minor decreases with the number of countries in which it becomes established (Fig. 4). The probability that the impact severity of a species will be Moderate remains approximately constant, regardless of the number of countries in which it is established, a result of the high variation in geographic range across this subset of species (Fig. 4).

### 3.4 Islands and impact severity

Accounting for both distance to the equator (*p* = 0.1775) and landmass area (*p* = 0.6407) did not improve model fit, with the optimal model containing landmass type as the only fixed effect whilst retaining species identity as the random effect. Nevertheless, landmass type had no significant effect on impact severities when categorized as harmful or not (*p* = 0.5). The proportion of harmful to not harmful species was equivalent across landmass types (Table 3). This result was characterized, however, by high random effect variance (*σ^2^* = 1.223), demonstrating both the unequal research effort and range of impact assessment outcomes across insect species.

**Table 3:** Comparison of harmful environmental impact information between island and mainland landmasses. Harmful impacts are those characterized as impact severities that range from being responsible for population declines to the irreversible extinction of native species (i.e., include Moderate (MO), Major (MR) and Massive (MV) categories). There was no difference in the proportion of impact studies that had evidence of harmful impacts between island and mainland landmasses. Information was based on all environmental impact evidence, i.e., some species were included more than once.

	Island	Mainland
Total number of species	53	94
Total number of impact studies	154	384
Impact severity frequency: MC, MN, MO, MR, MV	23, 78, 46, 7, 0	54, 200, 110, 20, 0
No. harmful impacts (percentage of total)	53 (34%)	130 (34%)
Mean number of studies per species (sd)	3.28 (σ = 4.00)	4.59 (σ = 7.40)
Median number of studies per species	1	3

## 4 Discussion

### 4.1 Biogeography of environmental impact

Biogeographical knowledge of the environmental impacts of alien insects is vital, particularly because the environmental impacts of individual invasive alien species, as the results here clearly show, are not homogenous across their introduced ranges (Jeschke et al., 2014). For example, *A. gracilipes* and *P. megacephala* had evidence of environmental impact ranging from minor to major, depending on the geographical region. In spite of this context dependence, a novel generality that emerged from the analysis we conducted is that the probability of an alien insect impact leading to local native species extinction (i.e. having a Major impact) increases with the number of countries in which it is established. Similarly, the probability of a species being associated with less severe impacts (Minimal Concern and Minor) declined with an increase in introduced range. There are three possible explanations, and related hypotheses in invasion biology, for this finding: (i) the broader the global introduced range of a species, the more likely it will be to have a severe impact somewhere within that introduced range, e.g., empty niche (MacArthur, 1970) and opportunity windows hypotheses (Johnstone, 1986); (ii) Species with severe impacts have (on average) larger invaded ranges because of some intrinsic biological property, e.g., ideal weed hypothesis (Baker, 1965; Rejmánek and Richardson, 1996); (iii) Knowledge shortfalls and the uneven spread of research attention given to alien insect species and across different insect orders create biases that lead to high profile species being preferentially observed. Our results provide direction for addressing current knowledge shortfalls by revealing those species and taxonomic groups of most in need, relatively speaking, of research attention to enable improved inferences. Extending this need to occurrence information would also enable the use of metrics such as extent of occurrence (EOO) or area of occupancy (AOO) to be used for range size calculations (Hughes et al. 2021; Latombe et al. 2022).

The evidence presented here suggests that while not all widely introduced alien insects have severe impacts, those that do tend to be more widely introduced. Exceptions to this may be due to a stronger influence of the other dimensions of environmental impact. Teasing apart each dimension (i.e. range, abundance and per capita effect) to identify the primary drivers of impact and provide a more information rich impact score would be valuable, although such analyzes will be data-intensive (Latombe et al. 2022). The multiplicative nature of the early model for measuring environmental impact proposed by Parker et al. (1999), Impact = Range x Abundance x *Per capita* effect, implies that the environmental impact of a species could be largely a function of a species abundance and/or its per capita effect (Bradley et al., 2019; Parker et al., 1999), rather than its range size. For example, the parasitic larvae of the fly *Philornis downsi* are largely responsible for the population decline of multiple endemic bird species in the Galapagos archipelago (Bulgarella et al., 2018). This insect is causing irreparable damage, yet it is only known to have alien populations in two locations, the Galapagos and Brazil (Bulgarella et al., 2018). In other words, this fly has a severe local impact, but a narrow, introduced range. In fact, our results show that the probability that an alien insect causes a decline in a native population is independent of the extent of its introduced range. Although IAS country inventories continue to improve (Pagad et al. 2022), a concerted effort is needed to strategically reduce biogeographical gaps in information on impact knowledge improving confidence in range size - impact relationships.

Islands are broadly considered to be hotspots for IAS driven extinctions (Bellard et al., 2016; Blackburn et al., 2004; Doherty et al., 2016; Reaser et al., 2007; Spatz et al., 2017), and as a result represent one of the most studied ecosystem types for the environmental impacts of IAS (Crystal-Ornelas and Lockwood, 2020). At least seven insect species assessed here have caused severe impacts on islands around the globe, including the local extinction of native species (LaPolla et al., 2000) and changes to entire ecosystem structure (O'Dowd et al., 2003). However, island ecosystems overall were no more likely to suffer from the harmful impacts of invasive insects than mainland locations, demonstrating a general lack of support for the island susceptibility hypothesis for invasive alien insects. This result is consistent with a similar finding for a small number of species and orders (16 species, 4 orders) (Cameron et al., 2016), and now provides comprehensive support (114 insect species across 11 orders) for a lack of island susceptibility for invasive insects.

### 4.2 Mechanisms and severities of environmental impact

Competitive interactions were the most prevalent form of environmental impact. This was particularly prominent within the Hymenoptera that outcompete natives via both interference (Boyce et al., 2003; Plentovich et al., 2018) and exploitative (Drescher et al., 2011; Hingston and Wotherspoon, 2017) competition. For some species outcompeting natives was the mechanism responsible for their most severe impacts in which native species were driven to local extinction (*Anoplolepsis gracilipes, Linepithema humile, Myrmica rubra, Pachycondyla chinensis, Paratrechina longicornis, Pheidole megacephala, Solenopsis invicta, Apis mellifera, Apis mellifera scutellata*) (Table S2). Only two non-Hymenopteran species (*Chrysomya albiceps* (Calliphoridae, Diptera) and *Digitonthophagus gazella* (Scarabaeidae, Coleoptera)) caused impacts of equivalent severity through competition (Hanski, 1977; Mesquita Filho et al., 2018).

In invasion biology, predatory impacts by invasive alien species are considered to be the leading mechanism causing native species decline (Doherty et al., 2016; Nunes et al., 2019). For example, by preying upon a native wingless fly, the carabid beetle *Merizodus soledadinus* is likely responsible for the fly's local extinction on the Kerguelen Islands (Lebouvier et al. 2020). Similarly, the big-headed ant (*Pheidole megacephala*) and the little fire ant (*Wasmannia auropunctata*) are both recorded as preying upon native species to the point of local extinction in Hawaii (LaPolla et al., 2000) and Gabon (Dunham and Mikheyev, 2010), respectively. Here for insects specifically, consumer-resource interactions including both predation and herbivory were only the second most prevalent and severe impact mechanisms after competition. Herbivorous insects are similarly responsible for severe environmental impacts, the most notable of which is the hemlock woolly adelgid (*Adelges tsugae*) driven mortality of large sections of eastern hemlock along the eastern coast of the U.S.A. (Abella, 2017). Two other species, the cottony cushion scale (*Icerya purchasi*) and the cycad aulacaspis scale (*Aulacaspis yasumatsui*), are similarly responsible for severe herbivory impacts on native flora in Guam and the Galapagos Archipelago (Marler and Krishnapillai, 2020; Roque-Albelo et al., 2003).

The largest proportion of recorded impacts were by Hymenoptera, both in terms of total number of species with evidence and total number of instances of impact evidence. This is despite orders such as Coleoptera and Hemiptera having more introduced species of environmental concern (GRIIS, Pagad et al. 2022) and Coleoptera, Lepidoptera and Diptera being more speciose in general (Stork, 2018). The Hymenoptera include some of the most well studied invasive insects, including the red imported fire ant and the Argentine ant (Crystal-Ornelas and Lockwood, 2020; Kenis et al., 2009). To date, species of the less well studied Blattodea, Dermaptera, Siphonaptera, and Thysanoptera have only minor or minimal environmental impact. There are two reasons why the maximum impact severity of a species may be assessed as low; either (i) there is an absence of evidence for more severe impacts, or (ii) there is evidence that the species is not having more severe impacts, i.e. evidence of an absence of an effect (Clarke et al., 2021). Most often it is the former rather than the latter, suggesting that a precautionary approach to IAS prioritization and management would be wise until evidence of an absence of impact has been gathered for each species.

### 4.3 Why are there so many data deficient species?

Geographical biases in IAS impact research are well known (Bellard and Jeschke, 2016; Crystal-Ornelas and Lockwood, 2020; Kenis et al., 2009; Pyšek et al., 2008). Here, for example, 59% of environmental impact evidence occurred within the Americas IPBES region, of which 83% occurred within North America. Indeed, the IPBES subregions of North America, Western Europe, and Oceania accounted for 75% of all recorded environmental impact evidence. However, countries within these subregions also generally have most recorded insect introductions. Nevertheless, even countries within these subregions with a relatively large number of known alien insects have pronounced impact research gaps (Bellard and Jeschke, 2016). For example, in the GRIIS country checklist, Denmark has at least 43 insect species recorded as introduced, but only one of these species (*Harmonia axyridis*) with evidence of environmental impact in that country (Howe et al., 2015; Howe et al., 2016). Similarly, many species only have impact evidence from a small subset of countries within their introduced range. For example, *H. axyridis* is one of the most widespread and commonly well-known alien insect species, with introduced populations in at least 45 countries, yet has evidence of environmental impact in only 14 (∼ 30%). That is, there is a large research deficit on the environmental impacts of alien insects from both a country and species perspective (Bellard and Jeschke, 2016).

Given the many known alien insect populations worldwide, and the importance of understanding and managing the environmental impacts caused by these populations, why is there such a dearth of information on environmental impacts for these species? Evans et al. (2018) concluded that environmental impact knowledge gaps for alien bird populations were not randomly distributed. Factors such as short residence time, small relative brain size and small alien geographic range were identified as being associated with data deficient species (Evans et al., 2018). Here, similar non-random distributions were identified for insects, with the proportion of species classed as Data Deficient differing according to taxonomic order. All species within the orders Embioptera, Ephemeroptera, Neuroptera, Odonata, Orthoptera, Phasmatodea, and Zygentoma were classed as Data Deficient, and only three orders, Blattodea, Hymenoptera, and Mantodea had more species with evidence of impact than without.

Three general reasons are proposed for why a given alien species may be lacking information on environmental impacts. The first is a general lack of interest in a species, or that it is perceived as an insufficient environmental threat to warrant concern and subsequent research (Evans et al., 2018; Pyšek et al., 2008). Research focus on a species is somewhat dictated by the public's awareness and interest in that species (Novoa et al., 2017). As such, species not placed on official IAS lists are likely to be overlooked in a management context (Uchida et al., 2016). Results of impact assessments such as we have conducted here may help overcome this phenomenon. Another possible reason is a species status as a known socioeconomic pest (Vilà et al., 2010). Impacts such as damage to food crops leading to decreased yields and monetary losses (Bradshaw et al., 2016; Fried et al., 2017; Paini et al., 2016) and the spread of disease (Mazza et al., 2014) capture the attention of the public and industry and tend, therefore, to be relatively better funded than environmental impact research. For example, the socioeconomic impacts of invasive alien gastropods are preferentially studied over their environmental impacts (Kesner and Kumschick, 2018). However, although such socioeconomic pests attract more research in general (Kumschick, Bacher, et al., 2015; McGeoch et al., 2015), we found, when accounting for research effort, an inverse relationship between socioeconomic pest status and the amount of environmental impact evidence. Furthermore, even within the topic of socioeconomic impacts there remain large gaps in our knowledge (Angulo et al., 2021; Renault et al., 2022). Finally, the perception of a species as an environmental threat leads to management interventions that may prevent species impacts from being realized. Prevention is the preferred IAS management approach as the feasibility for successful eradication decreases with an increase in the range or population size of an introduced species (Alvarez and Solís, 2018; Pluess et al., 2012; Tobin et al., 2014). As such, for some species there may be a discrepancy between the realized and potential impact severity, with a high potential sometimes leading to a smaller realized impact than expected. For example, the citrus long-horned beetle (*Anoplophora chinensis*), primarily known for its effects on fruit trees (Hérard and Maspero, 2019), is also perceived as an environmental threat to native species yet was classed as data deficient due to the perceived threat warranting enough concern for intervention.

The outcomes of an impact assessment for a particular species are invariably context specific – both in terms of when in the invasion process the impact assessment is conducted, as well as the ecosystem relevance of available or used evidence. As a result, care needs to be taken when using and communicating the results of such assessments. For example, if a given insect species is assessed as having a Moderate impact, depending on the quantity of evidence on the species this could apply to a specific alien population, within a specific spatial and temporal context and often only refers to a single native species being affected. One example of this is the moth *Lymantria dispar* where, depending on impact location and the species affected, has had a negative, neutral, or positive effect (Bell and Whitmore, 1997; Kasbohm et al., 1996). Meaningful comparisons of assessment results among insect species is also made difficult by the high variability in study designs used to record environmental impact evidence (Kumschick et al., 2015). For example, study duration can vary greatly, from months to years. The importance, and dearth, of studies examining the long-term impacts of invasive species has also been identified more generally (Strayer et al., 2006). Long-term observations may also reveal, for example, boom-bust population dynamics that can influence the management strategies for the species, as well as a potential mismatch between an impact severity score and the ongoing and ultimate environmental impact of an invasive species (Strayer et al., 2017). Such dynamics have been observed in insects, particularly in invasive ants (Cooling and Hoffmann, 2015; Cooling et al., 2018; Lester and Gruber, 2016; Mbenoun Masse et al., 2019). Another consideration is the different methods employed to determine the effect of invasive species on the native environment. Appropriate comparisons of environmental impacts require the use of a similar study design (Kumschick et al., 2015). One common method is to compare invaded and uninvaded sites, inferring impact from negative species co-occurrences. Depending on the design, this can potentially overestimate impact severity by treating native species absences as evidence of mortality as opposed to emigration (Human and Gordon, 1996). Regardless, it has been argued that negative species co-occurrences alone cannot conclusively lead one to infer invasive alien species as the proximal cause of the observed negative co-occurrence (Wauters et al., 2016), where the IAS could simply be a passenger of change rather than a cause (Bauer, 2012).

## 5 Conclusions

Using the most comprehensive available evidence, we find support for a relationship between alien geographic range size and environmental impact severity and the presence of taxonomic biases in research effort. However, we found no support for a bias toward insect species of socioeconomic concern nor did our results support the island susceptibility hypothesis. A focus on testing environmental impact-specific hypotheses in invasion biology and for insects specifically could advance such understanding. Mixed results for insects compared to other taxa were somewhat expected given that context dependence is pervasive in invasion ecology (Catford et al. 2021). However, searching for general phenomena in an apparently context-dependent area is also hindered by the narrow taxonomic and geographic coverage of environmental impact evidence offered by our current knowledge base. The ability to develop predictions and generalize results requires an even spread of knowledge across species, space and time, thus limiting the “poster child” effect (Watkins et al. 2021). A more strategic approach to collecting and synthesizing evidence of environmental impact would benefit national and local management scale prioritization for invasive alien insects, targeting priority information gaps such as underexplored species and areas, and testing theory to identify generalities. Here we provide a baseline of evidence and associated impact assessments to guide such efforts.

## CRediT authorship contribution statement

**David A. Clarke:** Conceptualization, Investigation, Data curation, Formal analysis, Writing – original draft. **Melodie A. McGeoch:** Conceptualization, Investigation, Writing – review & editing.

## Declaration of Competing Interest

The authors declare that they have no known competing financial interests or personal relationships that could have appeared to influence the work reported in this paper.

## Data Availability

All data and R code for replicating the analyzes can be found online (https://doi.org/10.5281/zenodo.7508641).

## Appendix Supplementary materials

Image, application 1

## References

[bib0001] Abella S.R. (2017). Forest decline after a 15-year “perfect storm” of invasion by hemlock woolly adelgid, drought, and hurricanes. Biol. Invasions.

[bib0002] Alvarez S., Solís D. (2018). Rapid response lowers eradication costs of invasive species. Choices.

[bib0003] Angulo E., Diagne C., Ballesteros-Mejia L., Adamjy T., Ahmed D.A., Akulov E., Banerjee A.K., Capinha C., Dia C.A.K.M., Dobigny G., Duboscq-Carra V.G., Golivets M., Haubrock P.J., Heringer G., Kirichenko N., Kourantidou M., Liu C., Nuñez M.A., Renault D., Roiz D., Taheri A., Verbrugge L.N.H., Watari Y., Xiong W., Courchamp F. (2021). Non-english languages enrich scientific knowledge: the example of economic costs of biological invasions. Sci. Total Environ..

[bib0004] Baker, H.G. (1965). Characteristics and modes of origin of weeds. In H. G. Baker & G.L. Stebbins (eds.), The genetics of colonizing species. (pp. 147-168).

[bib0005] Barbet-Massin M., Salles JM., Courchamp F. (2020). The economic cost of control of the invasive yellow-legged Asian hornet. NeoBiota.

[bib0006] Bates D., Maechler M., Bolker B., Walker S. (2015). Fitting linear mixed-effects models using lme4. J. Stat. Softw..

[bib0007] Bauer J.T. (2012). Invasive species: “back-seat drivers” of ecosystem change?. Biol. Invasions.

[bib0008] Beck J., Böller M., Erhardt A., Schwanghart W. (2014). Spatial bias in the GBIF database and its effect on modeling species' geographic distributions. Ecol. Inform..

[bib0009] Beggs J.R., Brockerhoff E.G., Corley J.C., Kenis M., Masciocchi M., Muller F., Rome Q., Villemant C. (2011). Ecological effects and management of invasive alien Vespidae. BioControl.

[bib0010] Bell J.L., Whitmore R.C. (1997). Eastern towhee numbers increase following defoliation by gypsy moths. Auk.

[bib0011] Bellard C., Cassey P., Blackburn T.M. (2016). Alien species as a driver of recent extinctions. Biol. Lett..

[bib0012] Bellard C., Jeschke J.M. (2016). A spatial mismatch between invader impacts and research publications. Conserv. Biol..

[bib0013] Blackburn T.M., Cassey P., Duncan R.P., Evans K.L., Gaston K.J. (2004). Avian extinction and mammalian introductions on oceanic islands. Science.

[bib0014] Blackburn T.M., Essl F., Evans T., Hulme P.E., Jeschke J.M., Kuhn I., Kumschick S., Markova Z., Mrugala A., Nentwig W., Pergl J., Pysek P., Rabitsch W., Ricciardi A., Richardson D.M., Sendek A., Vila M., Wilson J.R., Winter M., Genovesi P., Bacher S. (2014). A unified classification of alien species based on the magnitude of their environmental impacts. PLoS Biol..

[bib0015] Blackburn T.M., Pysek P., Bacher S., Carlton J.T., Duncan R.P., Jarosik V., Wilson J.R., Richardson D.M. (2011). A proposed unified framework for biological invasions. Trends Ecol. Evol..

[bib0016] Boyce W.M., O'Brien C.S., Rubin E.S. (2003). Response of bighorn sheep (Ovis canadensis) to feral honey bees (Apis mellifera) at water. Southwest. Nat..

[bib0017] Bradley B.A., Laginhas B.B., Whitlock R., Allen J.M., Bates A.E., Bernatchez G., Diez J.M., Early R., Lenoir J., Vilà M., Sorte C.J.B. (2019). Disentangling the abundance–impact relationship for invasive species. Proc. Natl. Acad. Sci..

[bib0018] Bradshaw C.J., Leroy B., Bellard C., Roiz D., Albert C., Fournier A., Barbet-Massin M., Salles J.M., Simard F., Courchamp F. (2016). Massive yet grossly underestimated global costs of invasive insects. Nat. Commun..

[bib0019] Bulgarella M., Quiroga M.A., Heimpel G.E. (2018). Additive negative effects of Philornis nest parasitism on small and declining Neotropical bird populations. Bird Conserv. Int..

[bib0020] Cameron E.K., Vilà M., Cabeza M. (2016). Global meta-analysis of the impacts of terrestrial invertebrate invaders on species, communities and ecosystems. Glob. Ecol. Biogeogr..

[bib0021] Carboneras C., Genovesi P., Vilà M., Blackburn T.M., Carrete M., Clavero M., D'Hondt B., Orueta J.F., Gallardo B., Geraldes P., González-Moreno P., Gregory R.D., Nentwig W., Paquet J.Y., Pyšek P., Rabitsch W., Ramírez I., Scalera R., Tella J.L., Walton P., Wynde R., Bieber C. (2018). A prioritised list of invasive alien species to assist the effective implementation of EU legislation. J Appl Ecol.

[bib0022] Cassey P., Delean S., Lockwood J.L., Sadowski J.S., Blackburn T.M. (2018). Dissecting the null model for biological invasions: a meta-analysis of the propagule pressure effect. PLoS Biol..

[bib0023] Catford J.A., Wilson J.R., Pyšek P., Hulme P.E., Duncan R.P. (2021). Addressing context dependence in ecology. Trends Ecol Evol.

[bib0024] Christensen, R.H.B. (2019). Ordinal – regression models for ordinal data. R package version 2019.12-10. https://CRAN.R-project.org/package=ordinal.

[bib0025] Clarke D.A., Palmer D.J., McGrannachan C., Burgess T.I., Chown S.L., Clarke R.H., Kumschick S., Lach L., Liebhold A.M., Roy H.E., Saunders M.E., Yeates D.K., Zalucki M.P., McGeoch M.A. (2021). Options for reducing uncertainty in impact classification for alien species. Ecosphere.

[bib0026] Cooling M., Hoffmann B.D. (2015). Here today, gone tomorrow: declines and local extinctions of invasive ant populations in the absence of intervention. Biol. Invasions.

[bib0027] Cooling M.D., Hoffmann B.D., Gruber M.A.M., Lester P.J. (2018). Indirect evidence of pathogen-associated altered oocyte production in queens of the invasive yellow crazy ant, Anoplolepis gracilipes, in Arnhem Land, Australia. Bull. Entomol. Res..

[bib0028] Crystal-Ornelas R., Lockwood J.L. (2020). The ‘known unknowns’ of invasive species impact measurement. Biol. Invasions.

[bib0029] Doherty T.S., Glen A.S., Nimmo D.G., Ritchie E.G., Dickman C.R. (2016). Invasive predators and global biodiversity loss. Proc. Natl. Acad. Sci. U. S. A..

[bib0030] Drescher J., Feldhaar H., Blüthgen N. (2011). Interspecific aggression and resource monopolization of the invasive ant anoplolepis gracilipes in Malaysian Borneo. Biotropica.

[bib0031] Dunham A.E., Mikheyev A.S. (2010). Influence of an invasive ant on grazing and detrital communities and nutrient fluxes in a tropical forest. Divers. Distrib..

[bib0032] Enders M., Havemann F., Ruland F., Bernard-Verdier M., Catford J.A., Gómez-Aparicio L., Haider S., Heger T., Kueffer C., Kühn I., Meyerson L.A., Musseau C., Novoa A., Ricciardi A., Sagouis A., Schittko C., Strayer D.L., Vilà M., Essl F., Hulme P.E., van Kleunen M., Kumschick S., Lockwood J.L., Mabey A.L., McGeoch M.A., Palma E., Pysek P., Saul W., Yannelli F.A., Jeschke J.M. (2020). A conceptual map of invasion biology: Integrating hypotheses into a consensus network. Glob. Ecol. Biogeogr..

[bib0033] Essl F., Hulme P.E., Jeschke J.M., Keller R., Pyšek P., Richardson D.M., Saul W.C., Bacher S., Dullinger S., Estévez R.A., Kueffer C., Roy H.E., Seebens H., Rabitsch W. (2016). Scientific and normative foundations for the valuation of alien-species impacts: thirteen core principles. Bioscience.

[bib0034] Evans T., Kumschick S., Blackburn T.M., Strubbe D. (2016). Application of the environmental impact classification for alien taxa (EICAT) to a global assessment of alien bird impacts. Divers. Distrib..

[bib0035] Evans T., Pigot A., Kumschick S., Şekercioğlu Ç.H., Blackburn T.M. (2018). Determinants of data deficiency in the impacts of alien bird species. Ecography.

[bib0036] Fortuna T.M., Le Gall P., Mezdour S., Calatayud P.A. (2022). Impact of invasive insects on native insect communities. Curr. Opin. Insect Sci..

[bib0037] Fried G., Chauvel B., Reynaud P., Sache I., Vilà M., Hulme P.E. (2017).

[bib0038] Gaston K.J. (1991). How large is a species' geographic range?. Oikos.

[bib0039] Genovesi P., Carboneras C., Vilà M., Walton P. (2014). EU adopts innovative legislation on invasive species: a step towards a global response to biological invasions?. Biol. Invasions.

[bib0040] Gippet J.M., Liebhold A.M., Fenn-Moltu G., Bertelsmeier C. (2019). Human-mediated dispersal in insects. Curr. Opin. Insect Sci..

[bib0041] Gruber M.A.M., Santoro D., Cooling M., Lester P.J., Hoffman B.D., Boser C., Lach L. (2023). A global review of socioeconomic and environmental impacts of ants reveals new insights for risk assessment. Ecol. Appl..

[bib0042] Hanski I. (1977). Biogeography and ecology of carrion flies in the Canary Islands. Ann. Entomol. Fennici.

[bib0043] Hawkins C.L., Bacher S., Essl F., Hulme P.E., Jeschke J.M., Kühn I., Kumschick S., Nentwig W., Pergl J., Pyšek P., Rabitsch W., Richardson D.M., Vilà M., Wilson J.R., Genovesi P., Blackburn T.M. (2015). Framework and guidelines for implementing the proposed IUCN environmental impact classification for alien taxa (EICAT). Divers. Distrib..

[bib0044] Hérard F., Maspero M. (2019). History of discoveries and management of the citrus longhorned beetle, Anoplophora chinensis, in Europe. J. Pest Sci..

[bib0045] Hijmans, R., Garcia, N., & Wieczorek, J. (2010). GADM: database of global administrative areas. Version 3.6 (released May 6, 2018).[Online].

[bib0046] Hill M.P., Clusella-Trullas S., Terblanche J.S., Richardson D.M. (2016). Drivers, impacts, mechanisms and adaptation in insect invasions. Biol. Invasions.

[bib0047] Hingston A.B., Wotherspoon S. (2017). Introduced social bees reduce nectar availability during the breeding season of the Swift Parrot (Lathamus discolor). Pacific Conserv. Biol..

[bib0048] Hoffmann B.D., Broadhurst L.M. (2016). The economic cost of managing invasive species in Australia. NeoBiota.

[bib0049] Hortal J., de Bello F., Diniz-Filho J.A.F., Lewinsohn T.M., Lobo J.M., Ladle R.J. (2015). Seven shortfalls that beset large-scale knowledge of biodiversity. Annu. Rev. Ecol. Evol. Syst..

[bib0050] Howe A.G., Ransijn J., Ravn H.P. (2015). A sublethal effect on native Anthocoris nemoralis through competitive interactions with invasive Harmonia axyridis. Ecol. Entomol..

[bib0051] Howe A.G., Ravn H.P., Pipper C.B., Aebi A. (2016). Potential for exploitative competition, not intraguild predation, between invasive harlequin ladybirds and flowerbugs in urban parks. Biol. Invasions.

[bib0052] Hughes A.C., Orr M.C., Ma K., Costello M.J., Waller J., Provoost P., Yang Q., Zhu C., Qiao H. (2021). Sampling biases shape our view of the natural world. Ecography.

[bib0053] Human K.G., Gordon D.M. (1996). Exploitation and interference competition between the invasive Argentine ant, Linepithema humile, and native ant species. Oecologia.

[bib0054] Inoue M.N., Yokoyama J., Washitani I. (2007). Displacement of Japanese native bumblebees by the recently introduced Bombus terrestris (L.) (Hymenoptera: Apidae). J. Insect Conserv..

[bib0055] IUCN (2020).

[bib0056] Jeschke J.M. (2008). Across islands and continents, mammals are more successful invaders than birds. Divers. Distrib..

[bib0057] Jeschke J.M., Bacher S., Blackburn T.M., Dick J.T., Essl F., Evans T., Gaertner M., Hulme P.E., Kuhn I., Mrugala A., Pergl J., Pysek P., Rabitsch W., Ricciardi A., Richardson D.M., Sendek A., Vila M., Winter M., Kumschick S. (2014). Defining the impact of non-native species. Conserv. Biol..

[bib0058] Johnstone I.M. (1986). Plant invasion windows: a time based classification of invasion potential. Biol. Rev..

[bib0059] Juliano S.A., Lounibos L.P. (2005). Ecology of invasive mosquitoes: effects on resident species and on human health. Ecol. Lett..

[bib0060] Kasbohm J.W., Vaughan M.R., Kraus J.G. (1996). Effects of gypsy moth infestation on black bear reproduction and survival. J. Wildlife Manag..

[bib0061] Kassambara, A. (2021). rstatix: Pipe-friendly framework for basic statistical tests. R package version 0.7.0, https://CRAN.R-project.org/pacakge=rstatix.

[bib0062] Kenis M., Auger-Rozenberg M.A., Roques A., Timms L., Péré C., Cock M.J., Settele J., Augustin S., Lopez-Vaamonde C. (2009). Ecological effects of invasive alien insects. Biol. Invasions.

[bib0063] Kesner D., Kumschick S. (2018). Gastropods alien to South Africa cause severe environmental harm in their global alien ranges across habitats. Ecol. Evol..

[bib0064] Kulhanek S.A., Ricciardi A., Leung B. (2011). Is invasion history a useful tool for predicting the impacts of the world's worst aquatic invasive species?. Ecol. Appl..

[bib0065] Kumschick S., Bacher S., Evans T., Marková Z., Pergl J., Pyšek P., Vaes-Petignat S., van der Veer G., Vilà M., Nentwig W. (2015). Comparing impacts of alien plants and animals in Europe using a standard scoring system. J. Appl. Ecol..

[bib0066] Kumschick S., Gaertner M., Vilà M., Essl F., Jeschke J.M., Pyšek P., Ricciardi A., Bacher S., Blackburn T.M., Dick J.T., Evans T., Hulme P.E., Kühn I., Mrugała A., Pergl J., Rabitsch W., Richardson D.M., Sendek A., Winter M. (2015). Ecological impacts of alien species: quantification, scope, caveats, and recommendations. BioScience.

[bib0067] Lapin K., Bacher S., Cech T., Damjanić R., Essl F., Georges FI., Hoch G., Kavčič A., Koltay A., Kostić S., Lukić I., Marinšek A., Nagy L., Agbaba S.N., Oettel J., Orlović S., Poljaković-Pajnik L., Sallmannshofer M., Steinkellner M., Stojnic S., Westergren M., Zlatkovic M., Zolles A., de Groot M. (2021). Comparing environmental impacts of alien plants, insects and pathogens in protected riparian forests. NeoBiota.

[bib0068] LaPolla J.S., Otte D., Spearman L.A. (2000). Assessment of the effects of ants on Hawaiian crickets. J. Orthoptera Res..

[bib0069] Latombe G., Catford J.A., Essl F., Lenzner B., Richardson D.M., Wilson J.R., McGeoch M.A. (2022). GIRAE: a generalised approach for linking the total impact of invasion to species' range, abundance and per-unit effects. Biol. Invasions.

[bib0070] Lebouvier M., Lambret P., Garnier A., Convey P., Frenot Y., Vernon P., Renault D. (2020). Spotlight on the invasion of a carabid beetle on an oceanic island over a 105-year period. Sci. Rep..

[bib0071] Lester P.J., Gruber M.A. (2016). Booms, busts and population collapses in invasive ants. Biol. Invasions.

[bib0072] Liebhold A.M., Brockerhoff E.G., Garrett L.J., Parke J.L., Britton K.O. (2012). Live plant imports: the major pathway for forest insect and pathogen invasions of the US. Front. Ecol. Environ..

[bib0073] Liebhold A.M., Yamanaka T., Roques A., Augustin S., Chown S.L., Brockerhoff E.G., Pyšek P. (2016). Global compositional variation among native and non-native regional insect assemblages emphasizes the importance of pathways. Biol. Invasions.

[bib0074] Liebhold A.M., Yamanaka T., Roques A., Augustin S., Chown S.L., Brockerhoff E.G., Pyšek P. (2018). Plant diversity drives global patterns of insect invasions. Sci. Rep..

[bib0075] MacArthur R. (1970). Species packing and competitive equilibrium for many species. Theor. Popul. Biol..

[bib0076] Marler T.E., Krishnapillai M.V. (2020). Longitude, forest fragmentation, and plant size influence Cycas micronesica mortality following island insect invasions. Diversity.

[bib0077] Mazza G., Tricarico E., Genovesi P., Gherardi F. (2014). Biological invaders are threats to human health: an overview. Ethol. Ecol. Evol..

[bib0078] Mbenoun Masse P.S., Tindo M., Djieto-Lordon C., Mony R., Kenne M. (2019). Diversity of ant assemblages (Hymenoptera: Formicidae) in an urban environment in Cameroon during and after colonization of the area by Wasmannia auropunctata. Eur. J. Entomol..

[bib0079] McGeoch M.A., Genovesi P., Bellingham P.J., Costello M.J., McGrannachan C., Sheppard A. (2016). Prioritizing species, pathways, and sites to achieve conservation targets for biological invasion. Biol. Invasions.

[bib0080] McGeoch M.A., Lythe M.J., Henriksen M.V., McGrannachan C.M. (2015). Environmental impact classification for alien insects: a review of mechanisms and their biodiversity outcomes. Curr. Opin. Insect Sci..

[bib0081] Mesquita Filho W., Flechtmann C.A., Godoy W.A., Bjornstad O.N. (2018). The impact of the introduced Digitonthophagus gazella on a native dung beetle community in Brazil during 26 years. Biol. Invasions.

[bib0082] Noriega J.A., Hortal J., Azcárate F.M., Berg M.P., Bonada N., Briones M.J., Del Toro I., Goulson D., Ibanez S., Landis D.A (2018). Research trends in ecosystem services provided by insects. Basic Appl. Ecol..

[bib0083] Novoa A., Dehnen-Schmutz K., Fried J., Vimercati G. (2017). Does public awareness increase support for invasive species management? Promising evidence across taxa and landscape types. Biol. Invasions.

[bib0084] Nunes A.L., Fill J.M., Davies S.J., Louw M., Rebelo A.D., Thorp C.J., Vimercati G., Measey J. (2019). A global meta-analysis of the ecological impacts of alien species on native amphibians. Proc. Biol. Sci..

[bib0085] Nuñez M.A., Amano T. (2021). Monolingual searches can limit and bias results in global literature reviews. Nat. Ecol. Evol..

[bib0086] O'Dowd D.J., Green P.T., Lake P.S. (2003). Invasional ‘meltdown'on an oceanic island. Ecol. Lett..

[bib0087] Pagad S., Genovesi P., Carnevali L., Schigel D., McGeoch M.A. (2018). Introducing the global register of introduced and invasive species. Sci. Data.

[bib0088] Pagad S., Bisset S., Genovesi P., Groom Q., Hirsch T., Jetz W., Ranipeta A., Schigel D., Sica Y.V., McGeoch M.A. (2022). Country compendium of the global register of introduced and invasive species. Sci. Data.

[bib0089] Paini D.R., Sheppard A.W., Cook D.C., De Barro P.J., Worner S.P., Thomas M.B. (2016). Global threat to agriculture from invasive species. Proc. Natl. Acad. Sci. U. S. A..

[bib0090] Parker I.M., Simberloff D., Lonsdale W., Goodell K., Wonham M., Kareiva P., Williamson M., Von Holle B., Moyle P., Byers J. (1999). Impact: toward a framework for understanding the ecological effects of invaders. Biol. Invasions.

[bib0091] Pebesma E. (2018). Simple features for R: Standardised support for spatial vector data. R J..

[bib0092] Plentovich S., Russell T., Fejeran C.C. (2018). Yellow crazy ants (Anoplolepis gracilipes) reduce numbers and impede development of a burrow-nesting seabird. Biol. Invasions.

[bib0093] Pluess T., Cannon R., Jarošík V., Pergl J., Pyšek P., Bacher S. (2012). When are eradication campaigns successful? A test of common assumptions. Biol. Invasions.

[bib0094] Pyšek P., Richardson D.M., Pergl J., Jarošík V., Sixtová Z., Weber E. (2008). Geographical and taxonomic biases in invasion ecology. Trends Ecol. Evol..

[bib0095] Pyšek P., Hulme P.E., Simberloff D., Bacher S., Blackburn T.M., Carlton J.T., Dawson W., Essl F., Foxcroft L.C., Genovesi P., Jeschke J.M., Kühn I., Liebhold A.M., Mandrak N.E., Meyerson L.A., Pauchard A., Pergl J., Roy H.E., Seebens H., van Kleunen M., Vilà M., Wingfield M.J., Richardson D.M. (2020). Scientists' warning on invasive alien species. Biol. Rev..

[bib0096] Reaser J.K., Meyerson L.A., Cronk Q., De Poorter M., Eldrege L., Green E., Kairo M., Latasi P., Mack R.N., Mauremootoo J., O'Dowd D.J., Orapa W., Sastroutomo S., Saunders A., Shine C., Thrainsson S., Vaiutu L. (2007). Ecological and socioeconomic impacts of invasive alien species in island ecosystems. Environ. Conserv..

[bib0097] Rejmánek M., Richardson D.M. (1996). What attributes make some plant species more invasive?. Ecology.

[bib0098] Renault D., Angulo E., Cuthbert R.N., Haubrock P.J., Capinha C., Bang A., Kramer A.M., Courchamp F. (2022). The magnitude, diversity, and distribution of the economic costs of invasive terrestrial invertebrates worldwide. Sci. Total Environ..

[bib0099] Renault D., Hess M.C.M., Braschi J., Cuthbert R.N., Sperandii M.G., Bazzichetto M., Chabrerie O., Thiébaut G., Buisson E., Grandjean F., Bittebiere A., Mouchet M., Massol F. (2022). Advancing biological invasion hypothesis testing using functional diversity indices. Sci. Total Environ..

[bib0100] Roque-Albelo L., Causton C., Mieles A. (2003). Population decline of galapagos endemic lepidoptera on volcán alcedo (Isabela Island, Galapagos Islands, Ecuador): an effect of the introduction of the cottony cushion scale. Bull. L'Institut R. des Sci. Nat. Belgique.

[bib0101] Roy H.E., Rabitsch W., Scalera R., Stewart A., Gallardo B., Genovesi P., Essl F., Adriaens T., Bacher S., Booy O., Branquart E., Brunel S., Copp G.H., Dean H., D'hondt B., Josefsson M., Kenis M., Kettunen M., Linnamagi M., Lucy F., Martinou A., Moore N., Nentwig W., Nieto A., Pergl J., Peyton J., Roques A., Schindler S., Schönrogge K., Solarz W., Stebbing P.D., Trichkova T., Vanderhoeven S., van Valkenburg J., Zenetos A. (2018). Developing a framework of minimum standards for the risk assessment of alien species. J. Appl. Ecol..

[bib0102] Seebens H., Bacher S., Blackburn T.M., Capinha C., Dawson W., Dullinger S., Genovesi P., Hulme P.E., van Kleunen M., Kuhn I., Jeschke J.M., Lenzner B., Liebhold A.M., Pattison Z., Pergl J., Pysek P., Winter M., Essl F. (2021). Projecting the continental accumulation of alien species through to 2050. Glob. Chang. Biol..

[bib0103] Seebens H., Blackburn T.M., Dyer E.E., Genovesi P., Hulme P.E., Jeschke J.M., Pagad S., Pysek P., van Kleunen M., Winter M., Ansong M., Arianoutsou M., Bacher S., Blasius B., Brockerhoff E.G., Brundu G., Capinha C., Causton C.E., Celesti-Grapow L., Dawson W., Dullinger S., Economo E.P., Fuentes N., Guenard B., Jager H., Kartesz J., Kenis M., Kuhn I., Lenzner B., Liebhold A.M., Mosena A., Moser D., Nentwig W., Nishino M., Pearman D., Pergl J., Rabitsch W., Rojas-Sandoval J., Roques A., Rorke S., Rossinelli S., Roy H.E., Scalera R., Schindler S., Stajerova K., Tokarska-Guzik B., Walker K., Ward D.F., Yamanaka T., Essl F. (2018). Global rise in emerging alien species results from increased accessibility of new source pools. Proc. Natl. Acad. Sci. U. S. A..

[bib0104] Simberloff D. (2009). The role of propagule pressure in biological invasions. Annu. Rev. Ecol. Evol. Syst..

[bib0105] Snyder C., MacQuarrie C.J., Zogas K., Kruse J.J., Hard J. (2007). Invasive species in the last frontier: distribution and phenology of birch leaf mining sawflies in Alaska. J. For..

[bib0106] Sol D. (2000). Are islands more susceptible to be invaded than continents? Birds say no. Ecography.

[bib0107] Spatz D.R., Zilliacus K.M., Holmes N.D., Butchart S.H.M., Genovesi P., Ceballos G., Tershy B.R., Croll D.A. (2017). Globally threatened vertebrates on islands with invasive species. Sci. Adv..

[bib0108] Stork N.E. (2018). How many species of insects and other terrestrial arthropods are there on earth?. Annu. Rev. Entomol..

[bib0109] Strayer D.L., D'Antonio C.M., Essl F., Fowler M.S., Geist J., Hilt S., Jaric I., Johnk K., Jones C.G., Lambin X., Latzka A.W., Pergl J., Pysek P., Robertson P., von Schmalensee M., Stefansson R.A., Wright J., Jeschke J.M (2017). Boom-bust dynamics in biological invasions: towards an improved application of the concept. Ecol. Lett..

[bib0110] Strayer D.L., Eviner V.T., Jeschke J.M., Pace M.L. (2006). Understanding the long-term effects of species invasions. Trends Ecol. Evol..

[bib119] Sullivan G.M., Feinn R. (2012). Using effect size - or why the P value is not enough. J. Grad. Med. Educ..

[bib0112] Tobin P.C., Kean J.M., Suckling D.M., McCullough D.G., Herms D.A., Stringer L.D. (2014). Determinants of successful arthropod eradication programs. Biol. Invasions.

[bib0113] Troudet J., Grandcolas P., Blin A., Vignes-Lebbe R., Legendre F. (2017). Taxonomic bias in biodiversity data and societal preferences. Sci. Rep..

[bib0114] Uchida S., Mori H., Kojima T., Hayama K., Sakairi Y., Chiba S. (2016). Effects of an invasive ant on land snails in the Ogasawara Islands. Conserv. Biol..

[bib0115] Vilà M., Basnou C., Pyšek P., Josefsson M., Genovesi P., Gollasch S., Nentwig W., Olenin S., Roques A., Roy D. (2010). How well do we understand the impacts of alien species on ecosystem services? A pan-European, cross-taxa assessment. Front. Ecol. Environ..

[bib0116] Watkins H.V., Yan H.F., Dunic J.C., Côté I.M. (2021). Research biases create overrepresented “poster children” of marine invasion ecology. Conserv. Lett..

[bib0117] Wauters N., Dekoninck W., Hendrickx F., Herrera H.W., Fournier D. (2016). Habitat association and coexistence of endemic and introduced ant species in the Galápagos Islands. Ecol. Entomol..

[bib0118] Yang L.H., Gratton C. (2014). Insects as drivers of ecosystem processes. Curr. Opin. Insect Sci..

